# Changes in prenatal testing during the COVID-19 pandemic

**DOI:** 10.3389/fped.2022.1064039

**Published:** 2022-11-09

**Authors:** Sara C. Handley, Rachel Ledyard, Lisbet S. Lundsberg, Molly Passarella, Nancy Yang, Moeun Son, Kathryn McKenney, Jay Greenspan, Kevin Dysart, Jennifer F. Culhane, Heather H. Burris

**Affiliations:** ^1^ Divison of Neonatology, Children’s Hospital of Philadelphia, Philadelphia, PA, United States; ^2^ Department of Pediatrics, Perelman School of Medicine at the University of Pennsylvania, Philadelphia, PA, United States; ^3^ Leonard Davis Institute of Health Economics, Philadelphia, PA, United States; ^4^ Department of Obstetrics, Gynecology, and Reproductive Services, Yale School of Medicine, New Haven, CT, United States; ^5^ Department of Obstetrics & Gynecology, University of Colorado, Aurora, CO, United States; ^6^ Division of Neonatology, Nemours duPont Pediatrics, Philadelphia, PA, United States; ^7^ Sidney Kimmel Medical College, Thomas Jefferson University, Philadelphia, PA, United States

**Keywords:** pregnancy, prenatal care, ultrasound, glucose tolerance test, gestational diabetes, access to care, COVID-19

## Abstract

**Objective:**

The coronavirus disease 2019 (COVID-19) pandemic disrupted healthcare delivery, including prenatal care. The study objective was to assess if timing of routine prenatal testing changed during the COVID-19 pandemic.

**Methods:**

Retrospective observational cohort study using claims data from a regional insurer (Highmark) and electronic health record data from two academic health systems (Penn Medicine and Yale New Haven) to compare prenatal testing timing in the pre-pandemic (03/10/2018–12/31/2018 and 03/10/2019–12/31/2019) and early COVID-19 pandemic (03/10/2020–12/31/2020) periods. Primary outcomes were second trimester fetal anatomy ultrasounds and gestational diabetes (GDM) testing. A secondary analysis examined first trimester ultrasounds.

**Results:**

The three datasets included 31,474 pregnant patients. Mean gestational age for second trimester anatomy ultrasounds increased from the pre-pandemic to COVID-19 period (Highmark 19.4 vs. 19.6 weeks; Penn: 20.1 vs. 20.4 weeks; Yale: 18.8 vs. 19.2 weeks, all *p* < 0.001). There was a detectable decrease in the proportion of patients who completed the anatomy survey <20 weeks' gestation across datasets, which did not persist at <23 weeks' gestation. There were no consistent changes in timing of GDM screening. There were significant reductions in the proportion of patients with first trimester ultrasounds in the academic institutions (Penn: 57.7% vs. 40.6% and Yale: 78.7% vs. 65.5%, both *p* < 0.001) but not Highmark. Findings were similar with multivariable adjustment.

**Conclusion:**

While some prenatal testing happened later in pregnancy during the pandemic, pregnant patients continued to receive appropriately timed testing. Despite disruptions in care delivery, prenatal screening remained a priority for patients and providers during the COVID-19 pandemic.

## Introduction

The coronavirus disease 2019 (COVID-19) pandemic created many disruptions in healthcare delivery, including obstetric care. Changes to obstetric care delivery were made quickly to decrease the risk of virus transmission across inpatient and outpatient settings (1, 2). Health systems and clinics responded by transitioning to or increasing virtual visits and adopting reduced visit schedules (3, 4). However, some aspects of prenatal care are not amenable to virtual care encounters. Essential services, such as the obstetric ultrasound to assess fetal anatomy and gestational diabetes screening, require in-person interactions (5, 6). The ability of health systems to provide, and pregnant patients to access these essential, in-person obstetric services in a timely manner during the COVID-19 pandemic is unclear.

To date, much of the literature regarding restructuring prenatal care in the setting of the COVID-19 pandemic has focused on telehealth visits and tailoring prenatal care schedules based on a pregnant patient's risk profile (7, 8). Studies have reported efforts to align in-person visits with essential obstetric testing, yet the frequency with which such testing was completed during the height of the pandemic is rarely described (3). There are also reports of combining ultrasound-based tests (e.g., first trimester dating ultrasound with an ultrasound to measure nuchal translucency) and examining completion of third trimester testing for HIV, syphilis, and routine urine collection as a marker of the adequacy of prenatal care (2, 9). Yet, despite the importance of second trimester testing in the ongoing management of a pregnancy and associated implications for the infant, from identification of birth defects to glucose monitoring after birth, the frequency and timing of such testing during the pandemic remains unknown.

The objective of this study was to assess if the timing of essential prenatal testing changed between the pre-pandemic and the early COVID-19 pandemic periods. We examined two second trimester services as primary outcomes the timing of (1) ultrasound for fetal anatomy and (2) gestational diabetes screening with a glucose tolerance test (GTT) or glucose challenge test (GCT). We examined receipt of first trimester ultrasound as a secondary outcome. Given the disruption and strain the COVID-19 pandemic created in the health care system, we hypothesized that routine screening would happen later in pregnancy during the COVID-19 pandemic.

## Methods

### Study design and population

This was a retrospective observational cohort study using claims data from an insurer in the MidAtlantic and electronic health record (EHR) data from two academic health systems to compare prenatal screening during the COVID-19 pandemic (03/10/2020–12/31/2020) with the pre-pandemic period (matched months in the two years prior; 03/10/2018–12/31/2018 and 03/10/2019–12/31/2019). This study was approved by the Children's Hospital of Philadelphia, University of Pennsylvania, and Yale University Institutional Review Boards.

Given the geographic, socioeconomic, and racial and ethnic differences in SARS-CoV-2 infection, hospitalizations, and deaths, analyses utilized data from three different and complementary sources to increase study generalizability (10–13). Insurer data came from Highmark, an independent licensee of the Blue Cross Blue Shield Association, that provided insurance coverage to people living in all of Delaware, southwestern Ohio (one county), across Pennsylvania (63 of 67 counties), and all of West Virginia during the study period. These data have geographic variation across metropolitan and non-metropolitan areas. The two health systems studied were Penn Medicine and Yale New Haven Hospital. The Penn Medicine health system serves the greater Philadelphia area, which spans southeastern Pennsylvania and central New Jersey, a major metropolitan region with racial, ethnic, and socioeconomic diversity. The Yale New Haven Health system provides care for the smaller metropolitan center of New Haven and the surrounding areas of Connecticut whose population composition is different than that in Philadelphia.

Pregnant patients included in the primary cohort, which was created to examine primary outcomes (second trimester testing), met al.l three of the following inclusion criteria: (1) <14 weeks' gestation by 03/10/2020 (last menstrual period 12/04/2019–3/9/2020); (2) gave birth at ≥20 weeks' gestation by 12/31/2020; and (3) singleton pregnancies. We used an analytic dataset from Highmark that required ZIP code to be non-missing. In order to capture appropriate prenatal care, further inclusion criteria depended on the data source. In those insured by Highmark, patients had to be enrolled in a plan by before 14 weeks' gestation. Patients at Penn Medicine and Yale New Haven Health systems had to have initiated prenatal care, either in-person or *via* telemedicine, before 28 weeks' gestation. The analytic dataset was checked to ensure that no pregnant patients were in both the Highmark and Penn Medicine data. A secondary cohort of pregnant patients, which was created to examine receipt of first trimester ultrasound, included pregnant patients who were <5 weeks' gestation by 03/10/2020 (last menstrual period 02/02/2020–03/09/2020), and met the same birth and prenatal care initiation criteria as the primary cohort.

### Study outcomes

The primary outcomes were the timing of essential second trimester testing: (1) ultrasound to assess fetal anatomy (e.g., “anatomy scan”, “full fetal survey”) and (2) glucose tolerance testing (GTT) or glucose challenge testing (GCT) to screen for gestational diabetes. The second trimester of pregnancy included the period from 14 weeks and 0 days to 27 weeks and 6 days. Timing was assessed by the number of completed weeks' gestation. In addition to timing, the proportion of pregnant patients completing testing before 20 weeks' given potential implications for pregnancy management and by the recommended time point before 23 weeks' gestation for second trimester ultrasound and before 29 weeks' gestation for GTT/GCT was assessed.

The second trimester ultrasound to assess fetal anatomy was identified using current procedural terminology (CPT) codes 76805, 76810, 76811, 76812, 76813, 76815, and 76816 in the Highmark data, EHR procedure names “Ultrasound complete” and “US Preg 2nd/3rd tri” occurring at ≥14 weeks' gestation in the Penn Medicine data, and in the Yale New Haven data the aforementioned CPT codes with the addition of 76801 and 76802 for ultrasounds specified as “complete”. If pregnant patients had more than one ultrasound to fully assess fetal anatomy, the first ultrasound to assess fetal anatomy was used to examine timing. The identification of GTT/GCT screening utilized CPT codes 82950, 82951, and 82952 in the Highmark data, EHR procedure names “1 h glucose gestational 1 h”, “2 h glucose tolerance - 2 h”, “2 h glucose tolerance 1 h”, “2 h glucose tolerance fasting”, “3 h glucose gestational 1 h”, “3 h glucose gestational 2 h”, “3 h glucose gestational 3 h”, “3 h glucose gestational fasting”, “Fasting glucose in glucose tolerance”, “Glucose tolerance test,$gestational,4spec(100 g)”, and “Glucose, gestational screen (50 g)-140 cutoff” in the Penn Medicine data, and all previously listed 1 and 3 h EHR procedure names in the Yale New Haven data. This definition considered the first GTT/GCT completed during the second trimester and did not differentiate between one-hour GCT or, two- or three-hour GTT.

The secondary outcome was receipt of first trimester ultrasound, which is routinely used to confirm an intrauterine pregnancy and provide an assessment of gestational age. This was defined as the first ultrasound of any type in the three data sources. The proportion of patients who completed a first trimester ultrasound before 14 weeks' gestation (when dating of a pregnancy is most accurate) was also examined.

### Study variables

Pregnant patient and area-level sociodemographic characteristics as well as pre-existing and pregnancy-associated conditions were assessed, given associations with disparities related to COVID-19 and potential risk factors for increased prenatal testing. These characteristics included age (<20, 20–<25, 25–<30, 30–<35, and ≥34 years) (14), race/ethnicity (examined as Asian, Hispanic, Non-Hispanic Black, Non-Hispanic White, and Another, Unknown or Missing), insurance type (private or public) (15), pregnant patient ZIP code of residence, nulliparity, smoking during pregnancy [defined in Highmark data using International Classification of Diseases (ICD-10) code O993 and current smoker, former smoker, or never smoker in the EHR], obesity (identified in the Highmark data using ICD-10 codes E660–E662, E664–E669, Z683, Z684, and O9921 and a pre-pregnancy body mass index ≥30 in the EHR data), pre-existing hypertension (defined in the Yale New Haven data with ICD-10 codes I10–I16 or O10 and in the Penn data as these ICD-10 codes on two or more occurrences at least 30 days apart), hypertensive disorders of pregnancy, including gestational hypertension (defined in the Yale data with ICD-10 codes O12 and O13 and in the Penn data as these ICD-10 codes on two or more occurrences at least one day apart), preeclampsia (defined in the Yale data with ICD-10 codes O11 and O14 and in the Penn data as these ICD-10 codes on two or more occurrences at least one day apart), Hemolysis, Elevated Liver enzymes and Low Platelets (HELLP) (defined using ICD-10 code O142), and eclampsia (defined using ICD-10 code O15), pre-existing diabetes (defined in the Yale data as ICD-10 codes E08-E11, E13, O240, O241, and O243 and in the Penn data these ICD-10 codes on two or more occurrences at least 30 days apart) (16), gestational diabetes (defined in the Yale data as ICD-10 code O244 and in the Penn data as this ICD-10 code on two or more occurrences at least one day apart) (17), preterm birth (defined as birth <37 weeks' gestation), and SARS-CoV-2 positivity during pregnancy. Race/ethnicity and nulliparity variables were not available in the insurer data. Patient ZIP code was not available in the Yale EHR data.

### Statistical analysis

Characteristics of patients across the three datasets were reviewed. Within each data source, bivariate analyses were used to compare pregnant patient sociodemographic characteristics and medical conditions in the pre-pandemic and the early COVID-19 pandemic periods. The timing of second trimester ultrasound to fully assess fetal anatomy, GTT/GCT, and initial first trimester ultrasound was similarly compared between periods. Bivariate tests of association were performed using *χ*^2^ or Fisher exact test as appropriate for categorical measures, and *t*-test or Wilcoxon for continuous measures. Multivariable logistic regression models were used to assess changes in testing timing between the two periods. Specifically, changes in second trimester ultrasound to assess fetal anatomy before 20 and 23 weeks' gestation, GTT/GCT before 29 weeks' gestation, and the receipt of a first trimester United States were assessed. Model adjustment included the following variables: maternal age, insurance type, obesity, smoking, pre-existing hypertension and pre-gestational diabetes. Pre-gestational diabetes was not included in models assessing GTT/GCT timing. Analyses were completed using SAS 9.4, Cary, NC.

## Results

Across the three data sources there were 31,474 pregnant patients included. Of those, 22,167 (70.4%) were patients from the Highmark cohort, 5,724 (18.2%) from the Penn Medicine health system, and 3,583 (11.4%) from the Yale New Haven health system. Pregnant patient sociodemographic characteristics and medical conditions are reported in Table 1. Pregnant patients in the Highmark data were primarily privately insured (91.8%), the plurality of pregnant patients in the Penn Medicine data were non-Hispanic Black (41.3%), and the rates of smoking during pregnancy (7.3%) and pre-existing hypertension (10.1%) were higher in the pregnant patients from Yale New Haven. Patient characteristics between the pre-pandemic and early COVID-19 pandemic periods were compared within each dataset (Supplementary Table S1). There were no consistent differences in patient characteristics between periods across the datasets.

**Table 1:** Patient characteristics in each of the three data sources.

Data source	Highmark	Penn Medicine	Yale New Haven
Total patients (n)	22,167	5,724	3,583
Birth year			
2018	7,954 (35.9%)	1,915 (33.5%)	1,227 (34.2%)
2019	7,404 (33.4%)	1,957 (34.2%)	1,192 (33.3%)
2020	6,809 (30.7%)	1,852 (32.4%)	1,164 (32.5%)
Sociodemographic characteristics			
Age (years)			
<20	595 (2.7%)	106 (1.9%)	84 (2.3%)
20–<25	3,340 (15.1%)	674 (11.8%)	398 (11.1%)
25–<30	6,501 (29.3%)	1,278 (22.3%)	864 (24.1%)
30–<35	7,462 (33.7%)	2,071 (36.2%)	1,304 (36.4%)
≥35	4,269 (19.3%)	1,595 (27.8%)	933 (26.0%)
Race and Ethnicity	Unavailable		
Hispanic	Unavailable	422 (7.4%)	775 (21.6%)
Non-Hispanic Asian	Unavailable	441 (7.7%)	199 (5.6%)
Non-Hispanic Black	Unavailable	2,363 (41.3%)	632 (17.6%)
Non-Hispanic White	Unavailable	2,220 (38.8%)	1,868 (52.1%)
Another/Unknown/Missing	Unavailable	278 (4.9%)	109 (3.0%)
Private Insurance	20,351 (91.8%)	3,488 (60.9%)	2,179 (60.8%)
Health characteristics			
Nulliparous	Unavailable	2,595 (45.3%)	1,484 (41.4%)
Smoked during pregnancy	1,028 (4.6%)	174 (3.0%)	263 (7.3%)
Obesity (BMI ≥30 kg/m^2^)	4,929 (22.2%)	1,544 (27.0%)	1,014 (28.3%)
Pre-existing HTN	928 (4.2%)	332 (5.8%)	363 (10.1%)
Any HDP	2,628 (12.1%)	1,147 (20.0%)	742 (20.7%)
Gestational HTN	1,512 (6.8%)	741 (13.0%)	442 (12.3%)
Preeclampsia	964 (4.4%)	390 (6.8%)	269 (7.5%)
HELLP	64 (0.3%)	13 (0.2%)	14 (0.4%)
Eclampsia	88 (0.4%)	3 (0.1%)	17 (0.5%)
Preexisting diabetes	296 (1.3%)	129 (2.3%)	79 (2.3%)
Gestational diabetes	1,872 (8.4%)	424 (7.4%)	295 (8.6%)
Preterm birth (<37 weeks’ gestation)	1,893 (8.5%)	502 (8.8%)	274 (7.7%)

BMI, body mass index; HTN, hypertension; HDP, hypertensive disorder of pregnancy; HELLP, Hemolysis, Elevated Liver enzymes and Low Platelets.

Hypertensive disorder of pregnancy includes gestational hypertension, preeclampsia, HELLP, and eclampsia.

**Figure 1:**
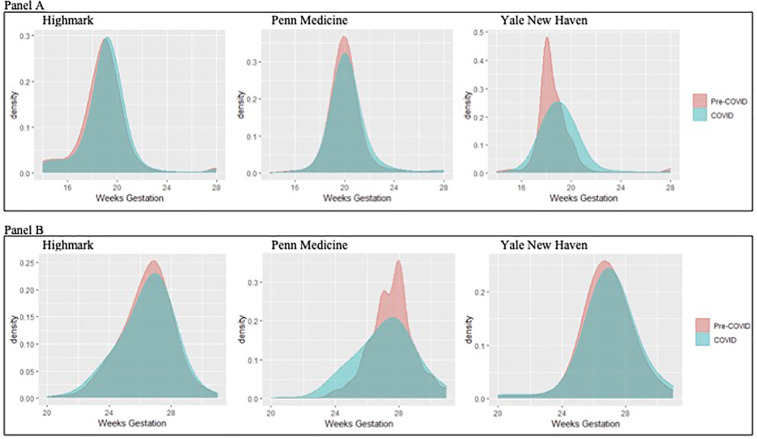
Distribution of completion of second trimester testing with an ultrasound to assess fetal anatomy (panel **A**) and gestational diabetes (panel **B**) across the three data sources.

The mean week of gestation for the initial second anatomy scan was significantly later in the COVID-19 period across all three datasets (Highmark: pre-pandemic 19.4 weeks vs. COVID-19 19.6 weeks *p* < 0.001; Penn Medicine: pre-pandemic 20.1 weeks vs. COVID-19 20.4 weeks *p* < 0.001; Yale New Haven: pre-pandemic 18.8 weeks vs. COVID-19 19.2 weeks *p* < 0.001). Figure 1 (panel A) illustrates the distribution of timing for completion of a second trimester ultrasound for fetal anatomy. The proportion of patients who completed second trimester ultrasound testing <20 weeks during the COVID-19 pandemic period was lower in all three datasets (Highmark: pre-pandemic 70.3% vs. COVID-19 64.2% *p* < 0.001; Penn Medicine: pre-pandemic 21.5% vs. COVID-19 15.9% *p* < 0.001; Yale New Haven: pre-pandemic 82.5% vs. COVID-19 63.8% *p* < 0.001). In the adjusted models, the odds of a second trimester ultrasound occurring ≥20 weeks' gestation in the COVID-19 period was significantly higher in all three datasets (Table 2). By 23 weeks' gestation, the proportion of pregnant patients who completed second trimester ultrasound screening had increased with no detectable difference in rates between pre-pandemic and COVID-19 periods across data sources (Highmark: pre-pandemic: 96.5% vs. COVID-19 96.6% *p* = 0.95, Penn Medicine: pre-pandemic: 95.1% vs. COVID-19 93.9% *p* = 0.08, Yale New Haven: pre-pandemic: 96.7% vs. COVID-19 95.8% *p* = 0.21), findings which were consistent in the adjusted analysis (Table 2).

**Table 2:** Unadjusted and adjusted odds of second trimester prenatal testing timing during the COVID-19 pandemic period.

Prenatal test	Highmark		Penn Medicine		Yale New Haven	
	Odds ratio (95% CI)	Adjusted odds ratio (95% CI)	Odds ratio (95% CI)	Adjusted odds ratio (95% CI)	Odds ratio (95% CI)	Adjusted odds ratio (95% CI)
First 2nd trimester fetal anatomy ultrasound ≥20 weeks	1.32 (1.24, 1.41)	1.33 (1.25, 1.41)	1.46 (1.25, 1.70)	1.46 (1.25, 1.71)	2.68 (2.28–3.14)	2.70 (2.29–3.17)
First 2nd trimester fetal anatomy ultrasound ≥23 weeks	0.98 (0.84, 1.15)	1.04 (0.89, 1.22)	1.27 (0.99, 1.64)	1.27 (0.98, 1.65)	1.27 (0.88–1.83)	1.30 (0.89–1.88)
First GCT/GTT ≥29 weeks	0.92 (0.82, 1.04)	1.00 (0.88, 1.12)	0.93 (0.80, 1.07)	0.91 (0.79, 1.06)	1.31 (1.03–1.66)	1.38 (1.08–1.76)

All models reference the pre-pandemic period. GCT/GTT models reference testing between 20 and 29 completed weeks’ gestation.

The mean week of gestation for which gestational diabetes testing was completed was similar between periods in the Highmark cohort and earlier in the academic institutions (Highmark: pre-pandemic 25.1 weeks vs. COVID-19 24.9 weeks *p* = 0.1; Penn Medicine: pre-pandemic 27.0 weeks vs. COVID-19 26.4 weeks *p* < 0.001; Yale New Haven: pre-pandemic 24.2 weeks vs. COVID-19 23.6 weeks *p* = 0.01). The distribution of timing of completion of gestational diabetes testing by gestation age week is shown in Figure 1 (panel B), for which there were no statistically significant changes in completion of timing before 29 weeks' gestation in the Highmark and Penn Medicine data (Highmark: pre-pandemic 90.9% vs. COVID-19 91.5% *p* = 0.21, Penn Medicine: pre-pandemic 75.5% vs. COVID-19 76.5% *p* = 0.45) and a decrease in the proportion of testing completed before 29 weeks' gestation in the Yale New Haven data (pre-pandemic 87.8% vs. COVID-19 84.7% *p* = 0.03). These results were consistent in the adjusted models (Table 2).

**Figure 2:**
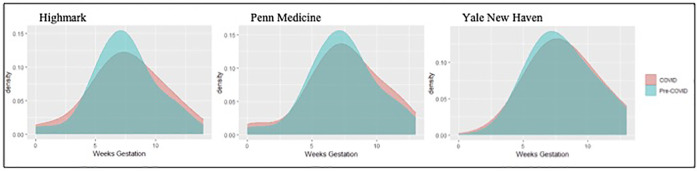
Distribution of completion of first trimester ultrasound across the three data sources.

Across the three data sources there were a total of 6,310 pregnant patients in the secondary cohort who were <5 weeks' gestation by 03/10/2020 for which the secondary outcome of first trimester ultrasound was examined. The distribution of timing for the initial ultrasound in the first trimester is shown in Figure 2. The proportion of pregnant patients who completed a first trimester ultrasound (before 14 weeks' gestation) was unchanged during the COVID-19 pandemic period for patients captured in the Highmark data (pre-pandemic 82.4% vs. 83.8%, *p* = 0.63), but decreased significantly among patients seen in the Penn Medicine and Yale New Haven health systems (Penn Medicine pre-pandemic 58.7% vs. COVID-19 41.0%, *p *= <0.001; Yale New Haven 78.8% vs. COVID-19 66.8%, *p *= <0.001). This finding in the academic institutions persisted in adjusted analyses, as the COVID-19 period was associated with higher odds of not completing a first trimester ultrasound (Penn Medicine: adjusted odds [aOR] 2.11, 95% confidence interval [CI] 1.74, 2.56, Yale New Haven: aOR 1.93, 95% CI 1.49, 2.51).

## Discussion

Although the average time at which second trimester ultrasounds occurred during the early phase of the COVID-19 pandemic was later in pregnancy, overall patients continued to receive appropriate routine prenatal second trimester ultrasounds and gestational diabetes testing. However, in both academic institutions the rate of receipt and adjusted odds of a first trimester ultrasound was significantly lower during the COVID-19 period. While our data demonstrate that prenatal testing during the second trimester in the COVID-19 period continued to meet guidelines from professional organizations, first trimester, in-person services may have been deprioritized.

The Guidelines for Perinatal Care recommend completion of an ultrasound to assess fetal anatomy between 18 and 22 weeks' gestation (18). While our data demonstrate there was no difference in the proportion of pregnant patients who completed this testing in the recommended time frame (before 23 weeks’ gestation), we did appreciate a shift in the mean gestational week during which this testing occurred and the proportion of scans completed before 20 weeks' gestation. The reason for this shift is likely multifactorial. First, prior to the pandemic, there were baseline practice differences across the three patient groups, with patients in the Yale New Haven system often receiving a second trimester ultrasound for fetal anatomy earlier in gestation than the other cohorts. Second, the shift in timing seen across the three datasets likely reflects systemic changes. For example, practices shifted scans to later in gestation to avoid incomplete image acquisition which requires additional in-person encounters increasing the risk of COVID-19 exposure. However, while screening was still completed as recommended, the shift in timing may have implications for pregnancies in which abnormal fetal anatomy is diagnosed. The detection of severe and potentially life-limiting congenital anomalies may influence a patient's decision to end a pregnancy which is often very time sensitive. Furthermore, associated diagnoses made during the second trimester may affect ongoing monitoring of complicated pregnancies and at-risk fetuses identified during second trimester ultrasound testing.

Our data regarding glucose tolerance testing was not consistent across the data sources, with a detectable shift only noted in the Yale New Haven data. This shift may reflect changes in coordination of in-person appointments and testing, which have been described in the literature at other academic institutions who were working to streamline appointments and decrease the number of contacts with the healthcare system. However, the overall timely completion of gestational diabetes testing likely reflects the dedication of providers and patients to ensure the timeliness of this testing, given the downstream effects on blood sugar management *via* dietary changes and medication initiation, which has implications for maternal, fetal, and neonatal wellbeing.

One of the more surprising findings was the change in receipt of a first trimester ultrasound, which was a prominent finding in the two academic health systems studied, but not in the Highmark data. It is important to consider potentially contributing factors. One factor may be the differences in sociodemographic characteristics between patients captured in the different data sources, specifically insurance type. A much higher percentage of the Highmark patients were privately insured, a characteristic associated with utilization of obstetric care and early initiation of prenatal care (19, 20). Another explanation is that the capture of first trimester ultrasounds is more complete in the insurer data as it reflects billable services outside of a single health system. In contrast, it is plausible that patients in the Penn Medicine or Yale New Haven cohorts were more likely to have their first trimester ultrasounds completed outside of these respective health systems, especially during the peak of the pandemic if patients perceived the burden of COVID-19 to be higher in tertiary health systems. However, the low rates of first trimester ultrasounds are concerning. First trimester ultrasounds are standardly used in conjunction with the last menstrual period to determine the gestational age of a pregnancy. Without an accurate last menstrual period or first trimester ultrasound, pregnancy dating is less accurate, which can have ramifications on pregnant patients and their infants both at the limits of viability as well as management of pregnancies that surpass their estimated due date. First trimester ultrasounds allow for the confirmation of an intrauterine pregnancy, identification of multiple gestation pregnancies, and diagnosis of cesarean scar pregnancies and other abnormalities or disorders that may affect the health of the pregnant patient, viability of the pregnancy, and associated monitoring. A first trimester ultrasound is also often a component of aneuploidy screening and may facilitate early diagnosis of severe anomalies (e.g., acrania). Timely diagnosis of severe pregnancy related abnormalities or complications and congenital anomalies during the first trimester is particularly relevant and may be time-sensitive given evolving access to abortion services in the United States.

This study has limitations. The three datasets did not have all the same variables available. For example, race and ethnicity and nulliparity were not available in the Highmark data. Similarly, not all variables were captured in the same way across the three datasets (i.e., use of CPT codes vs. EHR procedure names). Though we worked to harmonize the data as best as possible, these differences may contribute to variability across cohorts (i.e., rates of hypertension). We could not determine the reason for first trimester ultrasounds and could not differentiate between viability and dating ultrasounds. While changes in prenatal testing may have downstream effects on pregnant patients and their infants, we could not capture other outcomes in this study, such as changes in pregnancy monitoring or termination after ultrasounds nor the details of GDM management for the patient during pregnancy nor infant after birth. These and other outcomes warrant examination in future studies.

This study also has important strengths. We leveraged three different and complementary datasets to improve study generalizability with the representation of different populations living in different communities with varying medical conditions. These three datasets capture several practice patterns both within and between health systems and across different payer-mix groups. Where we observed consistency across datasets, findings are likely to be similar in other health systems and patient populations as well.

In summary, while ultrasound-based testing in the first two trimesters was done later in gestation during the early phase of the COVID-19 pandemic in 2020, recommended second trimester testing was largely completed as indicated. The changes in receipt of first trimester testing, specifically first trimester ultrasound, may have potential downstream effects on pregnant patients and warrants attention and further study. Although the COVID-19 pandemic created a massive stress on the health care system, prenatal health care delivery was generally maintained thanks to the dedication and resilience of providers and patients who continued to prioritize second trimester testing.

## Data Availability

The datasets obtained, generated, and analyzed for this study may be requested through a data use agreement with Highmark, Penn Medicine, or Yale New Haven Health. Requests to access these datasets should be directed to handleys@chop.edu.
