# Supplementary material for: Changes in prenatal testing during the COVID-19 pandemic
Source: Front Pediatr. 2022 Nov 9;10:1064039. doi: 10.3389/fped.2022.1064039 (PMC9682111; doi:10.3389/fped.2022.1064039)
Supplement: Supplementary file 1 [file Table1.docx]

| **Supplemental Table 1. Patient characteristics in each of the three data sources in the pre-pandemic and COVID-19 pandemic periods** | | | | | | | | | |
| --- | --- | --- | --- | --- | --- | --- | --- | --- | --- |
| **Data source** | Highmark | | | Penn Medicine | | | Yale New Haven | | |
| **Period** | Pre-pandemic | COVID-19 |  | Pre-pandemic | COVID-19 |  | Pre-pandemic | COVID-19 |  |
| Total patients (n) | 15,359 | 6,808 |  | 3,872 | 1,852 |  | 2,419 | 1,164 |  |
|  | n (%) | n (%) | p-value | n (%) | N (%) | p-value | n (%) | n (%) | p-value |
| Sociodemographic characteristics | | | | | | | | | |
| Age (years) |  |  | 0.24 |  |  | 0.80 |  |  | 0.18 |
| <20 | 415 (2.7%) | 180 (2.6%) |  | 66 (1.7%) | 40 (2.2%) |  | 60 (2.9%) | 24 (2.1%) |  |
| 20- <25 | 2,363 (15.4%) | 977 (14.4%) |  | 461 (11.9%) | 213 (11.5%) |  | 258 (10.7%) | 140 (12.0%) |  |
| 25- <30 | 4,520 (29.4%) | 1,981 (29.1%) |  | 867 (22.4%) | 411 (22.2%) |  | 609 (25.2%) | 255 (21.9%) |  |
| 30- <35 | 5,133 (33.2%) | 2,329 (34.2%) |  | 1,403 (36.2%) | 668 (36.1%) |  | 865 (35.8%) | 439 (37.7%) |  |
| >=35 | 2,928 (19.1%) | 1,341 (19.7%) |  | 1,075 (27.8%) | 520 (28.1%) |  | 627 (25.9%) | 306 (26.3%) |  |
| Race and Ethnicity | Unavailable | Unavailable |  |  |  | 0.57 |  |  | 0.45 |
| Hispanic | Unavailable | Unavailable |  | 277 (7.2%) | 145 (7.8%) |  | 513 (21.2%) | 262 (22.5%) |  |
| Non-Hispanic Asian | Unavailable | Unavailable |  | 306 (7.9%) | 135 (7.3%) |  | 142 (5.9%) | 57 (4.9%) |  |
| Non-Hispanic Black | Unavailable | Unavailable |  | 1,579 (40.8%) | 784 (42.3%) |  | 427 (17.6%) | 205 (17.6%) |  |
| Non-Hispanic White | Unavailable | Unavailable |  | 1,521 (39.3%) | 699 (37.7%) |  | 1,257 (52.0%) | 611 (52.5%) |  |
| Another/Unknown/  Missing | Unavailable | Unavailable |  | 189 (4.9%) | 89 (4.8%) |  | 80 (3.3%) | 29 (2.5%) |  |
| Private Insurance | 13,958 (90.9%) | 6,393 (93.9%) | <0.001 | 2,390 (61.7%) | 1,098 (59.3%) | 0.077 | 1,465 (60.6%) | 714 (61.3%) | 0.66 |
| Health characteristics | | | | | | | | | |
| Nulliparous | Unavailable | Unavailable |  | 1,745 (45.1%) | 850 (45.9%) | 0.63 | 1,016 (42.0%) | 468 (40.2%) | 0.45 |
| Smoked during pregnancy | 788 (5.1%) | 240 (3.5%) | <0.001 | 112 (2.9%) | 62 (3.4%) | 0.35 | 186 (7.7%) | 77 (6.6%) | 0.25 |
| Obesity (BMI >30 kg/m^2^) | 3,371 (22.0%) | 1,558 (23.0%) | 0.30 | 1,018 (26.3%) | 526 (28.4%) | 0.16 | 664 (27.5%) | 350 (30.1%) | 0.06 |
| Pre-existing HTN | 630 (4.1%) | 298 (4.4%) | 0.35 | 236 (6.1%) | 96 (5.2%) | 0.17 | 220 (9.5%) | 133 (11.4%) | 0.07 |
| Any HDP | 1,739 (11.6%) | 889 (13.3%) | <0.001 | 733 (18.9%) | 414 (22.4%) | 0.003 | 493 (20.4%) | 249 (21.4%) | 0.48 |
| Gestational HTN | 1,001 (6.5%) | 511 (7.5%) | 0.005 | 452 (11.7%) | 289 (15.6%) | <0.001 | 287 (11.9%) | 155 (13.3%) | 0.23 |
| Preeclampsia | 629 (4.1%) | 335 (4.9%) | 0.004 | 271 (7.0%) | 119 (6.4%) | 0.71 | 189 (7.8%) | 80 (6.9%) | 0.41 |
| HELLP | 42 (0.3%) | 22 (0.3%) | 0.48 | 8 (0.2%) | 5 (0.3%) | 0.58 | 9 (0.4%) | 5 (0.4%) | 0.78 |
| Eclampsia | 67 (0.44%) | 21 (0.31%) | 0.19 | 2 (0.05%) | 1 (0.05%) | 0.94 | 8 (0.3%) | 9 (0.8%) | 0.07 |
| Pre-existing diabetes | 195 (1.3%) | 101 (1.5%) | 0.20 | 86 (2.2%) | 43 (2.3%) | 0.81 | 50 (2.1%) | 29 (2.5%) | 0.36 |
| Gestational diabetes | 1,209 (7.9%) | 663 (9.7%) | <0.001 | 291 (7.5%) | 133 (7.2%) | 0.66 | 196 (8.1%) | 99 (8.5%) | 0.54 |
| Preterm birth <37 weeks’ gestation) | 1,280 (8.3%) | 613 (9.0%) | 0.10 | 348 (9.0%) | 154 (8.3%) | 0.40 | 177 (7.3%) | 97 (8.3%) | 0.28 |

Abbreviations: BMI – body mass index, HTN – hypertension, HDP – hypertensive disorder of pregnancy, HELLP – Hemolysis, Elevated Liver enzymes and Low Platelets

Hypertensive disorder of pregnancy includes gestational hypertension, preeclampsia, HELLP, and eclampsia
